# Supplementary material for: Fast evolution of SOS-independent multi-drug resistance in bacteria
Source: eLife. 2025 Jul 9;13:RP95058. doi: 10.7554/eLife.95058 (PMC12240585; doi:10.7554/eLife.95058)
Supplement: Supplementary file 1. — Other mutations detected in the ΔrecA resistant isolates. [file elife-95058-supp1.docx]

**Table S1. Other mutations detected in the *ΔrecA* resistant isolates**

| **Gene** | **Mutation** | **Genomic Position** | **Animo Acid Changes** |
| --- | --- | --- | --- |
| *stfE* | A>AGGTTTTCGAGAGC | 1209618 | p.Val13fs |
| *puuC* | T > G | 1363695 | p.Phe318Cys |
| *cpxA* | T > C | 4104711 | p.Thr89Ala |
