# Supplementary material for: Fast evolution of SOS-independent multi-drug resistance in bacteria
Source: eLife. 2025 Jul 9;13:RP95058. doi: 10.7554/eLife.95058 (PMC12240585; doi:10.7554/eLife.95058)
Supplement: Supplementary file 3. — Plasmids used in this study (Ghodke et al., 2019). [file elife-95058-supp3.docx]

**Table S3. Plasmids used in this study**

| **Plasmid** | **Features** | **Source** |
| --- | --- | --- |
| pJM1071-*recA* | Low copy vector with constitutive *recA* promoter and native *recA* RBS; *recA* cloned in pJM1071 between *NdeI/XbaI* | Lab stock (59) |
| pKD46 | Temperature sensitive replication (*repA101ts*); encodes lambda Red genes | Lab stock |
| pCP20 | Temperature sensitive origin of replication; encodes the FLP recombinase. | Lab stock |
