# Supplementary material for: Fast evolution of SOS-independent multi-drug resistance in bacteria
Source: eLife. 2025 Jul 9;13:RP95058. doi: 10.7554/eLife.95058 (PMC12240585; doi:10.7554/eLife.95058)
Supplement: Supplementary file 4. — Primers used in this study. [file elife-95058-supp4.docx]

**Table S4. Primers used in this study**

| **Name** | **Sequence** | **Function** |
| --- | --- | --- |
| *recA-FWD* | AAAAAAGCAAAAGGGCCGCAGATGCGACCCTTGTGTATCAAACAAGACGAGAAACGAGAGAGGATGCTCAC | Construction of *recA* deletion mutant |
| *recA-REV* | CAACAGAACATATTGACTATCCGGTATTACCCGGCATGACAGGAGTAAAAGACGTCTAAGAAACCATTATTATCATGAC |  |
