# Supplementary material for: Fast evolution of SOS-independent multi-drug resistance in bacteria
Source: eLife. 2025 Jul 9;13:RP95058. doi: 10.7554/eLife.95058 (PMC12240585; doi:10.7554/eLife.95058)
Supplement: Source code 1. [file elife-95058-code1.zip › 0-Matlab-STORM-eLife2025/gapolyfitndir/01554782.pdf]

The Use of a Genetic Algorithm to Optimize the Functional Form of a Multi-dimensional Polynomial Fit to Experimental Data

Janet Clegg
Department of Electronics
University of York.
York UK
jc@ohm.york.ac.uk

John F. Dawson
Department of Electronics
University of York.
York UK
jfd@ohm.york.ac.uk

Stuart J. Porter
Department of Electronics
University of York.
York UK
sjp@ohm.york.ac.uk

Mark H. Barley
ICI Strategic Technology Group.
Wilton Centre
Wilton UK
mark_barley@ici.com

Abstract- This paper begins with the optimisation of three test functions using a genetic algorithm and describes a statistical analysis on the effects of the choice of crossover technique, parent selection strategy and mutation. The paper then examines the use of a genetic algorithm to optimize the functional form of a polynomial fit to experimental data; the aim being to locate the global optimum of the data. Genetic programming has already been used to locate the functional form of a good fit to sets of data, but genetic programming is more complex than a genetic algorithm. This paper compares the genetic algorithm method with a particular genetic programming approach and shows that equally good results can be achieved using this simpler technique.

1 Introduction

The aim behind this work has been to locate the optimum of an objective function, based on a limited sampling of the function. The first part of the paper describes some initial work on the use of a simple genetic algorithm (GA) to optimise test functions chosen to be representative of the experimental data. The effects of the choice of crossover, parent selection, population size and mutation are analysed on these test functions.

Given a set of data it is often the case that some predictions are required. This may be a prediction of the optimal value or it may be required to interpolate for data values lying between those values already obtained. The method most frequently used to make these predictions is that of fitting a curve to the data and making these predictions from the fitted curve. In most cases the functional form of the fitted curve is pre-decided. The decision may be to fit a quadratic polynomial to the data and then use optimization techniques (usually least square methods) to find the best coefficients for this quadratic fit.

A common method used at present to derive information from a set of data is the "Design of Experiment" method (Montgomery 1997). In this method the experimental data is analysed by fitting a polynomial, usually to a small region of the data space, and then using response surface methods to estimate where the optimal value lies. The functions chosen to fit the data are predetermined polynomials that are either linear, quadratic or cubic. Not all data is suited to these particular functional fits, and if the fit is poor then poor predictions will be derived from these functions.

Genetic programming (Koza 1992, 1994) has been used recently to find a good functional fit to data (Davidson 1999) and genetic algorithms (GAs) have been used to locate the best parameter values in some pre-specified functional fit (Gulsen 1995, VanderNoot 1998). In this paper a GA (rather than genetic programming) is used to optimize the functional form of a polynomial fit to experimental data and a least square method is used to choose the coefficients for the polynomial.

Davidson (1999) used genetic programming to find a good polynomial fit to data, choosing the values of the coefficients in the polynomial with a least square algorithm. Genetic programming is more complex than a GA. The simple GA described here performs exactly the same task as Davidson's genetic program. Davidson verifies his technique by obtaining a polynomial fit to the Colebrook-White formula for friction in turbulent pipe flow. In this paper, it is demonstrated that a simple GA results in an equally good polynomial fit to the Colebrook-White formula. Both the Colebrook-White formula and the other test functions used in this paper do not involve any noise; future work will investigate the use of the method on noisy data.

Section 2 of this paper describes the initial work on the use of a simple GA to optimise three test functions. Section 3 describes details of the particular GA used to optimise the functional form of a polynomial fit to a given data set. Section 4 describes tests on the performance of this GA, by taking data from known functions and Section 5 compares the performance of this GA with the genetic programming technique by Davidson.

0-7803-9363-5/05/$20.00 ©2005 IEEE.

2 Statistical analysis of the GA

This section describes an analysis of the performance of a genetic algorithm when tested on three different functions that are representative of the experimental data. There are various selection strategies available for choosing parents, different techniques for performing crossover and various ways to mutate the population. It is not clear, when starting a new problem, which of these options are best. Since each run of a GA can be slightly different from the next, even when the options chosen remain the same, a statistical analysis must be carried out to obtain a reliable assessment of its performance. The GA has therefore been run many times to obtain its average performance. The number of runs necessary to obtain a good estimate of the average performance was assessed by performing a very large number of runs (1000 runs) and comparing the statistics from this with those obtained using smaller sample sizes. It was found that 50 runs gave a reasonable estimate of the statistics.

The statistical analysis of the GA has been repeated on three test problems and these are the optimization of the three functions given by equations (1)-(3). These functions are displayed in figures 1-3 respectively in two dimensions, although the analysis was performed on six dimensional versions of these functions. In the following figures and tables of results, the test problems will be referred to as functions 1-3. For all GA runs, the parameter values which are not varying are chosen to be flat crossover with tournament selection, population size 500 and a mutation rate m=10 (see section 2.3).

$$f(x) = \sum_{k=1}^{k=3} \frac{h_k}{b_k + r_k} \text{ with } r_k = \sqrt{\sum_{i=1}^{i=n} \frac{(c_{ki} - x_i)^2}{w_k}} \qquad (1)$$

$$f(x) = \sum_{k=1}^{k=3} h_k \exp(-r_k) \text{ with } r_k = \sum_{i=1}^{i=n} \frac{(c_{ki} - x_i)^2}{w_k} \qquad (2)$$

$$f(x) = -5\exp\left(-\frac{10}{n}\sqrt{\frac{1}{n}\sum_{i=1}^{i=n}(x_i - c_1)^2}\right) - \exp\left(\frac{1}{n}\sum_{i=1}^{i=n}\cos(b_1\pi(x_i - a_1)) + \frac{1}{n}\sum_{i=1}^{i=n}\sin(b_2\pi(x_i - a_2))\right) \qquad (3)$$

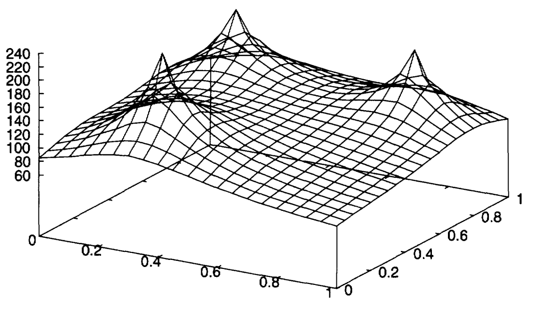

Figure 1. Function number 1

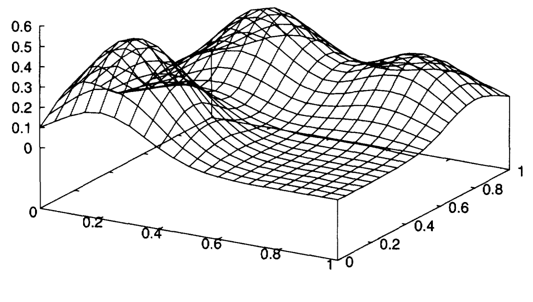

Figure 2. Function number 2

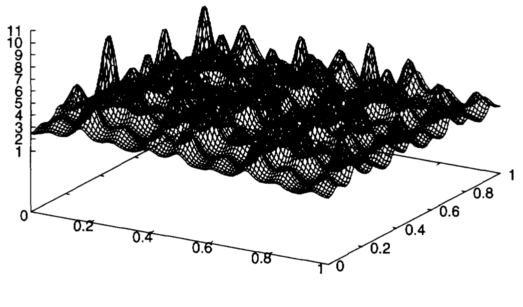

Figure 3. Function number 3

2.1 **Analysis of the Crossover Technique**

Five different crossover techniques have been investigated in this work. If the function to be optimized is of n variables and parent 1 has values $(p_1^1, p_2^1, \ldots, p_n^1)$, parent 2 is $(p_1^2, p_2^2, \ldots, p_n^2)$, child 1 is $(c_1^1, c_2^1, \ldots, c_n^1)$ and child 2 $(c_1^2, c_2^2, \ldots, c_n^2)$, then the following crossover techniques have been investigated.

Flat crossover (Radcliffe 1991)-Offpring are produced by choosing a uniformly random number $0 < r_i < 1$ and for k=1,2

$$c_i^k = p_i^1 + r_i * (p_i^2 - p_i^1) \quad \text{if } p_i^1 < p_i^2$$

Simple crossover (Wright 1991, Michalewicz 1992)- A random number $i \in \{1,2,\ldots n\text{-}1\}$ is chosen and two offspring are produced

$$(p_1^1,\ldots,p_i^1,p_{i+1}^2,\ldots,p_n^2) \text{ and } (p_1^2,\ldots,p_i^2,p_{i+1}^1,\ldots,p_n^1)$$

Arithmetic crossover (Michalewicz 1992)- A random number $r \in$ [-0.5,1.5] is chosen and offspring are produced

$$c_i^1 = r * p_i^1 + (1 - r) * p_i^2 \text{ and } c_i^2 = r * p_i^2 + (1 - r) * p_i^1$$

Discrete crossover (Muhlenbein 1993)- c_i is a randomly (uniformly) chosen value from the set $\{p_i^1, p_i^2\}$

BLX-α crossover (Eshelman 1993)- Offspring are produced where the value of c_i is a randomly (uniformly) chosen value in the interval $[p_{min} - \alpha \times I, p_{max} + \alpha \times I]$ where $p_{max} = \max(p_i^1, p_i^2)$, $p_{min} = \min(p_i^1, p_i^2)$ and $I = p_{max} - p_{min}$.

Table 1 contains the results of the statistical analysis of the performance of the crossover techniques described above. The table displays the generation numbers required (averaged over 50 runs) for the GA to reach its optimal value. It can be seen that flat crossover converges in the least number of generations for all the three test functions.

Crossover technique	Generations to optimum function 1	Generations to optimum function 2	Generations to optimum function 3
Flat	32	49	9
Simple	46	67	17
Arithmetic	68	95	22
Discrete	41	56	16
BLX-α	63	98	23

Table 1. Comparison of various crossover techniques

2.2 Analysis of Parent Selection Strategies

Five different parent selection strategies have been investigated and are described below.

Tournament selection - A random sample of k members of the population are selected and the member whose fitness function is best is chosen as a parent. Tournament selection has been tested for k = 2, 5, 10.

Roulette wheel selection - Each member P_i of the population is selected as parent with a probability of

$$\frac{f(P_i)}{\sum_{j=1}^{j=n} f(P_j)}$$

where f is the fitness function and n is the population size.

Elitist selection - Members of the population are sorted into order of fitness, and the best k members are chosen with equal probability as parents. Here we have chosen k =100.

Table 2 contains an analysis of the above selection strategies. The table displays the number of generations required (averaged over 50 runs of the GA) to reach the optimal value. The Roulette wheel strategy performs the poorest and the best performing strategy is Tournament selection with k =10.

Selection strategy	Generations to optimum function 1	Generations to optimum function 2	Generations to optimum function 3
Roulette	197	46	19
Tournament 2	33	47	9
Tournament 5	13	19	5
Tournament 10	11	18	4
Ellitist	13	21	5

Table 2. Various selection strategies and their performance

2.3 Analysis of the Mutation Rate

If a small mutation rate is chosen the GA will converge very quickly but is not guaranteed to converge to the global optimum. If, on the other hand, a large mutation rate is used the GA will more consistently converge to the global optimum but will take a very large number of generations in order to converge. The convergence rate of the GA can be improved by introducing a variable mutation rate (i.e. one that varies with generation number). In this paper a mutation rate has been used which is given by

$$\text{Probability that } x_i \text{ mutates} = \frac{m}{100} * \exp(-0.002882 * (n - 1))$$

where n=generation number. Figure 4 shows an example of how small, large and variable mutation rates perform in a GA. The x axis represents generation number and the y axis is the fitness function.

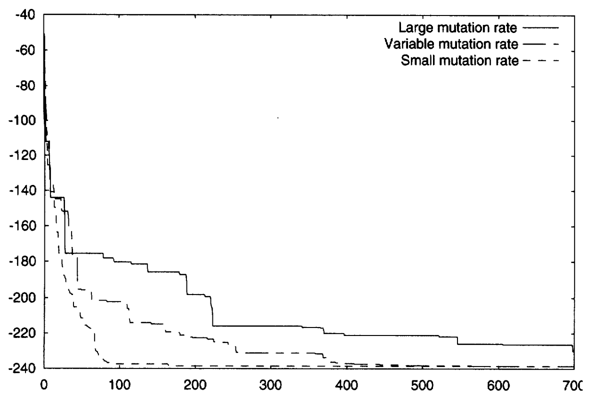

Figure 4. Comparison of convergence with mutation rate

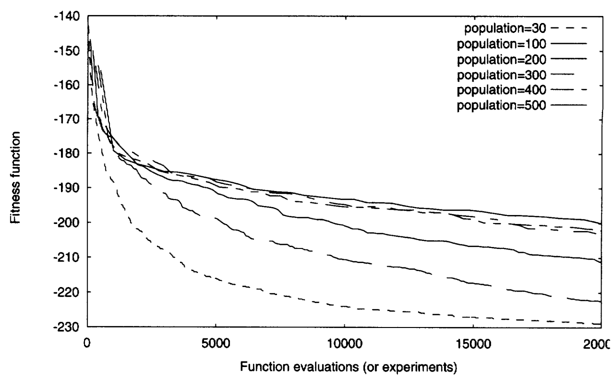

Figure 5. Convergence of GA with respect to number of fitness evaluations for different population sizes

Table 3 displays convergence rates (averaged over 50 runs) for the three test functions with different values of variable mutation rate *m*. The mutation rate *m*=10 was found to be the best performing, since this was the least value (and consequently the fastest converging) such that the global optimal was reached for all 50 runs of the GA. With *m*=4 some runs of the GA would converge to local optima rather than the global optimum.

Mutation rate *m*	Generations to optimum function 1	Generations to optimum function 2	Generations to optimum function 3
40	496	628	259
20	252	394	112
10	65	150	21
4	25	35	11

Table 3. Effects of mutation rate on converence of GA

2.4 Changing the population size

Increasing the population size in the GA means that more of the space is sampled at each generation; and therefore one might expect that fewer generations would be required to achieve convergence. But it does not necessarily follow that 10 generations of population size 100 performs the same as 20 generations of size 50 (i.e. convergence does not depend linearly on the number of evaluations of its population members). Figure 5 displays the fitness function plotted against the number of fitness evaluations with five different population sizes for function 1 (functions 2 and 3 behave similarly).

It can be seen that there are large differences in the convergence of the GA for population sizes 30, 100 and 200, whereas population sizes 300, 400 and 500 converge at very similar speeds. It can be deduced that there is not much to be gained from using a population size of 500, rather than one of 300.

3 Description of the GA for finding polynomial formula

In this section, a GA is described that is used to find the best polynomial fit to a set of data. The population in this particular GA consists of strings of integers, each string of integers representing one particular polynomial. A maximum power for each term in the polynomial and a maximum number of terms in the polynomial are pre-set before the GA begins. Each term in a general polynomial consists of a constant multiplying a term of the form

$$x_1^{p_1} x_2^{p_2} x_3^{p_3} \ldots x_n^{\ p_n}$$

where n is the number of variables and $\sum_{i=1}^{i=n} p_i$ must be less than or equal to the maximum power. Note that a constant term in the polynomial will have p_i=0 for all i. An example of how a particular polynomial is represented by a string of integers is given by the polynomial

$$a_1 x_1^5 x_2^2 x_3^1 + a_2 x_1^0 x_2^2 x_3^3 + a_3 x_1^1 x_2^7 x_3^0 + a_4 x_1^0 x_2^0 x_3^0$$

which has four terms and three variables. This polynomial is represented within the GA by the string of integers

{ (5,2,1), (0,2,3), (1,7,0), (0,0,0) }.

The cost function (or fitness function) in the GA is defined as the value of χ^2 after a linear least square fit has been performed to find the optimal values of the coefficients a_1, .. ,a_4.

Note that none of the terms in the polynomial should be the same since

$$a_1 x_1^5 x_2^2 x_3^1 + a_2 x_1^0 x_2^2 x_3^3 + a_3 x_1^1 x_2^7 x_3^0 + a_4 x_1^0 x_2^0 x_3^0 + a_5 x_1^5 x_2^2 x_3^1$$
$$= (a_1 + a_5) x_1^5 x_2^2 x_3^1 + a_2 x_1^0 x_2^2 x_3^3 + a_3 x_1^1 x_2^7 x_3^0 + a_4 x_1^0 x_2^0 x_3^0 \quad (4)$$

If a polynomial were represented with a repeat term as in the LHS of equation (4) the least square algorithm would be over specified and the equations in the algorithm would become singular. The least square software implements singular value decomposition and therefore would be able to deal with singularities such as these, but it is better to avoid this situation if possible.

The initial population is a set of randomly chosen polynomials. A random number is chosen to represent the number of terms in the polynomial (which must be less than the maximum number of terms) and random sequences of integers are chosen as the powers (with the restriction that their sum for each term must be less than the maximum power). If one term is a term already chosen for the current polynomial then it is rejected, since repeat terms are not allowed.

To describe the crossover technique used, consider that each parent consists of a selection of polynomial terms. Crossover should somehow randomly distribute the terms of the parents to terms in the offspring, i.e. offspring number 1 would have some of parent 1's terms and some of parent 2's terms, and likewise with offspring 2. The crossover technique must also take into account that repeated terms are not allowed.

Suppose the two selected parents are:-

{ (5,2,1), (0,2,3), (1,7,0), (0,0,0) } parent 1

and

{ (0,0,8), (0,2,3), (0,2,4), (3,0,1) } parent 2

then for each chromosome/term in parent 1 a random number between 0 and 1 is picked. For the first chromosome in parent 1, (5,2,1), suppose the random number picked is 0.75, then (5,2,1) will go to child 1 with probability 0.75 and child 2 with probability 0.25. This is repeated for all chromosomes in parent 1, so we may finish with offsprings

{ (0,2,3), (0,0,0), ... } child 1

{ (5,2,1), (1,7,0), ... } child 2.

The same procedure is repeated for the chromosomes of parent 2. When the chromosome (0,2,3) is picked from parent 2 (note that it already exists in child 1), probabilities are not used because repeat terms are not allowed, and it automatically goes to child 2.

Mutation is performed by randomly introducing a completely new term in the polynomial, checking that this particular term does not already exist. Variable mutation rate has been used with m=10 and population size 500.

4 Trial run of the GA for polynomial fits

The GA described in the previous section was tested using data sets taken from 100 randomly selected polynomials of two variables (the choice of two variables was to enable plotting of the surface in order to visually check the fit). For the 100 polynomials selected, the GA found a fitted polynomial whose χ^2 value was less than 1.0e-21 for 95 out of the 100 polynomials and for the remaining 5 polynomials, the value of χ^2 was no more than 1.2e-5. Therefore, even in the worst of the 100 test cases, the GA managed to find a very good fit.

The GA was also tested using a data set taken from the function $f(x, y) = \sin(5xy)$. The traditional way to approximate this function for x<1, y<1 is to use the series expansion of sin(x) and this would give us the polynomial approximation (using terms up to a power 7 in xy)

$$\sin(5xy) \approx 5xy - \frac{(5xy)^3}{3!} + \frac{(5xy)^5}{5!} - \frac{(5xy)^7}{7!}. \qquad (5)$$

After running the GA for its best polynomial fit, a polynomial was found that was a far better fit than the above traditional approximation in this range. The GA found the following polynomial fit

$$\sin(5xy)=-6.8x^2y^5-29.2x^2y^3-0.44xy^2-16.4x^3y^3-31.7x^3y^2+1.06xy^3+0.008+20.7x^2y^2+4.3xy+20.8x^4y^2+12.2x^4y^3+14.9x^3y^4+16.8x^2y^4-7.7x^5y^2+0.56x^3y. \qquad (6)$$

Figure 6 displays the function sin(xy), together with the series approximation in (5) and the polynomial fit from the GA given by (6). The series approximation is poor near the point x=1, y=1 and yet the polynomial produced by the GA is a good fit for all values of x and y.

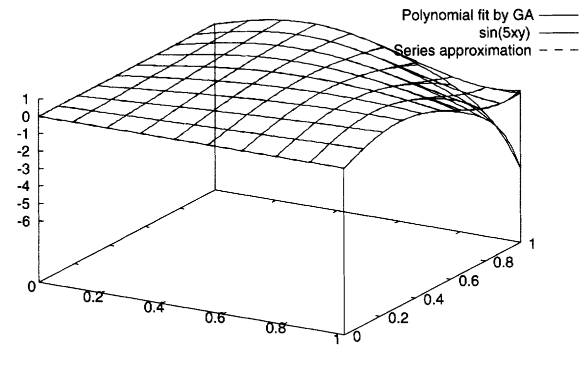

Figure 6. Series approximation to sin(xy) compared to polynomial fit from GA

As a further test the GA is used to locate the best polynomial fit to a radial basis type function as displayed in Figure 7. This is a good test for the GA since this function will be very difficult to fit with a polynomial, because of the sharp peaks.

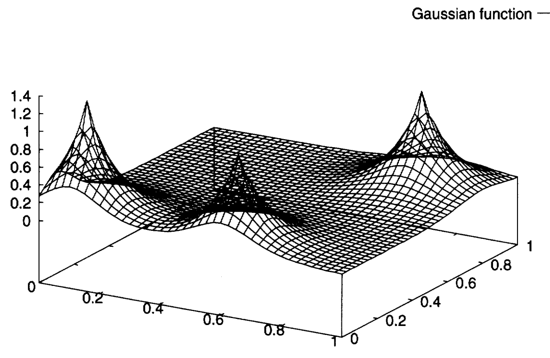

Figure 7. A radial basis type function

Figure 8 displays the polynomial fit from the GA allowing powers of up to 40, with maximum number of terms 100. It can be seen that the polynomial fit in Figure 8 is very good considering the type of surface it has to fit.

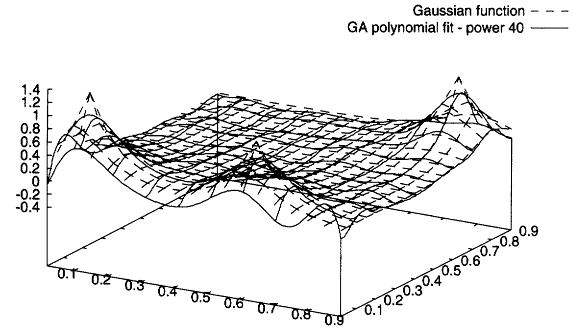

Figure 8. Optimal polynomial of degree 40 from the GA

By comparison, if we use the least square software on a polynomial consisting of *all* possible terms of a polynomial of order 40 (i.e. 1681 terms) to find the best possible coefficients we find that the best possible fit is as displayed in Figure 9.

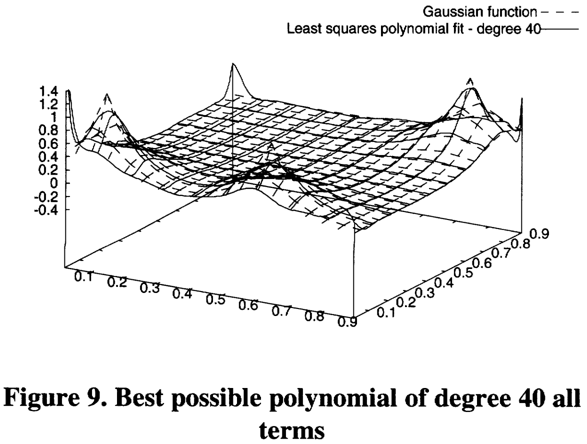

Figure 9. Best possible polynomial of degree 40 all terms

The GA has managed to find a polynomial which is almost as good a fit to the data using only 99 terms, compared to the least square best possible fit using all 1681 terms.

5 Comparison with a genetic programming technique

The idea of using evolutionary techniques to choose the terms of a polynomial fit and then performing a least square fit for its coefficients has been done before, except that genetic programming was used rather than a genetic algorithm. Davidson (1999) uses genetic programming to choose the terms of the fitting polynomial followed by a least square fit to find the best coefficients in that polynomial. He demonstrates his work by finding a polynomial approximation to the Colebrook-White formula that calculates the friction factor in a pipe depending on the Reynolds number and its relative roughness. Davidson's method follows from work by Babovic (1997a,b).

In genetic programming the members of the population are represented by trees in which each node of the tree is some operation (e.g. addition or multiplication). Davidson finds he needs to introduce an algorithm (called a rule-based component) operating on the tree in order to eliminate linear dependent terms and redundancies. He also manipulates the trees in the genetic program at each iteration in order to express power terms as multiple multiplications and to introduce a constant term. He adapts the normal method of crossover and mutation in a genetic program in order that each term in the polynomial is not split into separate parts.

Davidson's algorithm runs in a sequence of steps given by the following: (1) perform the rule-base component, (2) introduce a constant term, (3) perform a least square fit, (4) perform the rule base component a second time, (5) replace powers with multiplications in the tree and finally perform the adapted crossover and mutation. All these steps are repeated as the iterations of the genetic program proceed.

In order to compare our method with that of Davidson, we used our software to find its best polynomial fit to the Colebrook-White formula and compared it with Davidson's polynomial fit. To make comparisons, we look at the absolute difference between the value of the Colebrook-White formula and the polynomial approximation at the 100 data points, both the largest absolute difference and the sum of these absolute differences. The maximum absolute difference over the data points is 0.000194 for Davidson's polynomial and 0.000133 for our polynomial; the sum of the absolute differences is 0.00297 for Davidson and 0.00356 for our polynomial. So both Davidson's method and the method described in this paper give very similar

results, the difference being that the GA described here is far simpler.

6 Conclusions

This paper describes a statistical analysis of a simple genetic algorithm when tested on three different problems. Analysis is performed on various parent selection strategies, crossover techniques and methods of mutation.

A genetic algorithm has also been described which takes a set of data and searches for the best possible functional form of a polynomial fit to the data. The algorithm has been tested on various data sets and has also been compared to a genetic program implemented by Davidson (1999). It has been shown that the simple genetic algorithm described here locates an equally good fit to the Colebrook-White formula as that achieved by Davidson, who has used a more complex method. Future work will involve investigating the effects of noise within the data.

Acknowledgements

This work has been funded by the Imperial Chemical Industries PLC and Johnson Matthey PLC.

Bibliography

Babovic, V. and Abbott, M.B., "The evolution of equation from hydraulic data, Part I: Theory", *J. Hydraulic Res.*,35 ,1997a, 397-410.

Babovic, V. and Abbott, M.B., "The evolution of equation from hydraulic data, Part II: Applications", *J. Hydraulic Res.*, 35, 1997b, 411-430.

Babovic, V. and Keijzer, M., "Genetic programming as a model induction engine", *J. Hydroinformatics*, 2, 2000, 35-61.

Davidson, J. W., Savic, D., and Walters, G. A. "Method for the identification of explicit polynomial formulae for the friction in turbulent pipe flow". *ACM Journal of Hydroinformatics., 1,* (1999), 115-126.

Eshelman, L.J. and Schaffer, J.D., "Real-coded genetic algorithms and interval-schemata", *Foundation of genetic algorithms 2,* L. Darrell Whitley (Ed.) (Morgan Kaufmann Publishers, San Mateo), 1993, 187-202.

Gulsen, M., Smith, A. E. and Tate, D. M., "A genetic algorithm approach to curve fitting". *Int. J. Prod. Res.,* 33, no.7 (1995), 1911-1923.

Koza, J. R., "Genetic programming: On the Programming of Computers by Means of Natural Selection". Cambridge, 1992.

Koza, J.R.,"Genetic programming II: Automatic discovery of reusable programs". The MIT Press, 1994.

Michalewicz, Z., "Genetic algorithms + data structures = evolution programs", Springer-verlag, New York, 1992.

Montgomery, D. C., *Design and Analysis of Experiments.* Wiley 1997.

Muhlenbein, H. and Schlierkamp-Voosen, D., "Predictive models for the breeder genetic algorithm 1: Continuous parameter optimisation", *Evolutionary computation* 1, 1993, 25-49.

Radcliffe, N.J., "Equivalence class analysis of genetic algorithm", *Complex systems* 5, 1991, 183-205

VanderNoot, T. J. and Abrahams, I., The use of genetic algorithms in the non-linear regression of immittance data. *J. Electro. Chem.*, 448 part 1, (1998), 17-23.

Wright, A., "Genetic algorithms for real parameter optimisation", *Foundation of genetic algorithms* 1, G.J.E. Rawlin (Ed.)(Morgan Kaufmann, San Mateo), 1991, 205-218.
