# Supplementary material for: Fast evolution of SOS-independent multi-drug resistance in bacteria
Source: eLife. 2025 Jul 9;13:RP95058. doi: 10.7554/eLife.95058 (PMC12240585; doi:10.7554/eLife.95058)
Supplement: Source code 1. [file elife-95058-code1.zip › 0-Matlab-STORM-eLife2025/gridfitdir/demo/html/gridfit_demo.html]

gridfit\_demo


 


## Contents

- Topographic data
- Fitting a trigonometric surface
- The trig surface with highly different scalings on the x and y axes
- Fitting the "peaks" surface
- Using tiles in gridfit

```
% Gridfit demo script

% This script file is designed to be used in cell mode
% from the matlab editor, or best of all, use the publish
% to HTML feature from the matlab editor. Older versions
% of matlab can copy and paste entire blocks of code into
% the Matlab command window.
```

## Topographic data

```
load bluff_data;
x=bluff_data(:,1);
y=bluff_data(:,2);
z=bluff_data(:,3);
% Two ravines on a hillside. Scanned from a
% topographic map of an area in upstate New York.
plot3(x,y,z,'.')
```

```
% Turn the scanned point data into a surface
gx=0:4:264;
gy=0:4:400;
g=gridfit(x,y,z,gx,gy);
figure
colormap(hot(256));
surf(gx,gy,g);
camlight right;
lighting phong;
shading interp
line(x,y,z,'marker','.','markersize',4,'linestyle','none');
title 'Use topographic contours to recreate a surface'
```

## Fitting a trigonometric surface

```
clear

n1 = 15;
n2 = 15;
theta = rand(n1,1)*pi/2;
r = rand(1,n2);

x = cos(theta)*r;
y = sin(theta)*r;
x=x(:);
y=y(:);

x = [[0 0 1 1]';x;x;1-x;1-x];
y = [[0 1 0 1]';y;1-y;y;1-y];
figure
plot(x,y,'.')
title 'Data locations in the x-y plane'
```

```
z = sin(4*x+5*y).*cos(7*(x-y))+exp(x+y);

xi = linspace(0,1,51);
[xg,yg]=meshgrid(xi,xi);
zgd = griddata(x,y,z,xg,yg);

figure
surf(xi,xi,zgd)
colormap(hot(256))
camlight right
lighting phong
title 'Griddata on trig surface'
% Note the wing-like artifacts along the edges, due
% to the use of a Delaunay triangulation in griddata.
```

```
zgrid = gridfit(x,y,z,xi,xi);

figure
surf(xi,xi,zgrid)
colormap(hot(256))
camlight right
lighting phong
title('Gridfit to trig surface')
```

## The trig surface with highly different scalings on the x and y axes

```
xs = x/100;
xis = xi/100;

ys = y*100;
yis = xi*100;

% griddata has problems with badly scaled data
[xg,yg]=meshgrid(xis,yis);
zgd = griddata(xs,ys,z,xg,yg);

figure
surf(xg,yg,zgd)
colormap(hot(256))
camlight right
lighting phong
title 'Serious problems for griddata on badly scaled trig surface'

% autoscaling on (the default)
zgrids = gridfit(xs,ys,z,xis,yis,'autoscale','on');

% plot the autoscaled result
figure
surf(xis,yis,zgrids)
colormap(hot(256))
camlight right
lighting phong
title 'Gridfit (automatically scaled) on trig surface'
```

```
Warning: Duplicate x-y data points detected: using average of the z values.
```

 

## Fitting the "peaks" surface

```
clear

n = 100;
x = (rand(n,1)-.5)*6;
y = (rand(n,1)-.5)*6;

z = peaks(x,y);

xi = linspace(-3,3,101);
zpgf = gridfit(x,y,z,xi,xi);

[xg,yg]=meshgrid(xi,xi);
zpgd = griddata(x,y,z,xg,yg,'cubic');

figure
surf(xi,xi,zpgd)
colormap(jet(256))
camlight right
lighting phong
title 'Griddata (method == cubic) on peaks surface'

figure
surf(xi,xi,zpgf)
colormap(hsv(256))
camlight right
lighting phong
title('Gridfit to peaks surface')
```

 

## Using tiles in gridfit

```
% Users of gridfit who have really huge problems now have
% an option. I'll generate a large amount of data,
% and hope to model a fairly large grid - 800 x 800. This
% would normally require gridfit to solve a system of
% equations with 640,000 unknowns. It would probably be too
% large of a problem for my computer, were I to use gridfit
% on the full problem. Gridfit allows you to break the problem
% into smaller tiles if you choose. In this case each tile
% is 120x120, with a 25% (30 element) overlap between tiles.

% Relax, this demo may take a couple of minutes to run!!!!

n = 100000;
x = rand(n,1);
y = rand(n,1);
z = x+y+sin((x.^2+y.^2)*10);

xnodes = 0:.00125:1;
ynodes = xnodes;

[zg,xg,yg] = gridfit(x,y,z,xnodes,ynodes,'tilesize',120,'overlap',0.25);

surf(xg,yg,zg)
shading interp
colormap(jet(256))
camlight right
lighting phong
title 'Tiled gridfit'
```

Published with MATLAB® 7.0.1
