# Supplementary material for: Fast evolution of SOS-independent multi-drug resistance in bacteria
Source: eLife. 2025 Jul 9;13:RP95058. doi: 10.7554/eLife.95058 (PMC12240585; doi:10.7554/eLife.95058)
Supplement: Source code 1. [file elife-95058-code1.zip › 0-Matlab-STORM-eLife2025/gridfitdir/doc/Understanding_gridfit.rtf]

Understanding GridfitJohn R. D'Erricowoodchips@rochester.rr.com8/19/06IntroductionGridfit is a surface modeling tool, fitting a surface of the form z(x,y) to scattered (or regular) data. As it is not an interpolant, it allows the existence of replicates in your data with no problems. What do I mean by an "interpolant"? An interpolant is a code that is designed to always exactly predict all supplied data. Griddata and interp1 are examples of interpolants. Gridfit is more accurately described as an approximant. It produces a surface which represents the behavior of the supplied data as closely as possible, allowing for noise in the data and for replicate data.A nice feature of gridfit is its ability to smoothly extrapolate beyond the convex hull of your data, something that griddata cannot do (except by the slow, memory intensive 'v4' method.) Finally, gridfit does its extrapolation in a well behaved manner, unlike how polynomial models (for example) might behave in extrapolation. Gridfit also allows you to build a gridded surface directly from your data, rather than interpolating a linear approximation to a surface from a delaunay triangulation.This document describes the ideas behind gridfit.The mechanical and philosophical underpinnings of gridfitHow does gridfit work? Imagine a thin, flexible plate, attached to your data points by elastic bands. Each of these elastic bands draws the plate towards its corresponding data point, stretching only in the z direction. If your data points were to fall on a simple planar surface,	z(x,y) = a_0 + a_1 x + a_2 ythen the result will be a least squares plane. This works because the potential energy stored in a (perfect, linearly elastic) elastic band will be proportional to the square of its extension.Now imagine that the data points do not fall on a plane, but arise from some curved surface z(x,y). While the plate itself is flexible, I've allowed it some finite and non-zero bending rigidity that the user can control. The bands connecting the plate to our data points will pull the plate into a curved shape, while the bending rigidity of the plate resists deformation. The relative stiffness of the plate, as compared to the strength of the elastic bands will cause some tradeoff between fitting the data as well as possible and a smooth overall surface. It is this plate stiffness that allows gridfit to smoothly extrapolate beyond the data itself. Of course, extrapolation beyond the data will be well behaved, since the plate will tend to become locally planar wherever there is no data to deform it.The tradeoff between stiffness of the plate and the effective spring constant of the bands connecting the plate to the data allows the user to choose anything between the extremes of a purely planar fit, to a crinkly thing that can follow the bumps and hollows of noisy data.The methodology of gridfitFirst, lets look at how gridfit predicts a value at any location. Since the gridfit surface is defined by values at a set of nodes forming a rectangular lattice, any data point must lie in one of these rectangular cells of the lattice. (One requirement of gridfit is that the boundaries of its lattice must form a bounding box for all of the data supplied in the (x,y) plane.) Given a point inside a single rectangular cell of the lattice, there are three methods supplied in gridfit to impute a value at that point. They are:- Nearest neighbor interpolation- Triangle (linear) interpolation- Bilinear (tensor product linear) interpolation These methods were chosen to be simple to generate, as well as because they are very "local" methods.  They require no more than a few local values from the grid to predict the value at each point. This local nature makes the sparse linear least squares problem as efficient as possible, by keeping it maximally sparse.If we think of the surface that will result from gridfit as essentially a low order spline, it is easy to visualize the idea that interpolation at any point inside the grid is just a linear combination of the values at the grid nodes in the locality of the given point. Thus, we can write the interpolation problem in general as a linear algebra problem	A*x = ywhere the vector x is of length nx*ny (nx is the number of nodes in the x direction, and ny is the number of grid nodes in the y direction.) Thus A has n rows, corresponding to each data point supplied by the user, and nx*ny columns.It will not be atypical to expect that there will be more columns than rows in this "regression" problem. For example, a user with 100 data points might choose to build a gridded surface which has 100x100 nodes. The resulting regression problem will be massively underdetermined. As such, standard regression techniques, involving a solution like x = A\y or x = pinv(A)*y, will be inappropriate. The approach that gridfit takes, with its plate-like metaphor, is to solve a regularized problem. At every node of the surface, gridfit (using its default regularization method of 'gradient') attempts to force the (first) partial derivatives of the surface in neighboring cells to be equal. In effect, this results in a second set of linear equations of the form	B*x = 0where the derivatives are approximated using finite differences of the surface at neighboring nodes.The main alternative regularization method in gridit is the 'laplacian' method. It attempts to force the sum of second partial derivatives to zero. This is actually closely related to the gradient method, but with a subtly significant difference. Either method will work nicely. (The 'springs' regularizer for gridfit is a flawed one, at least in my humble opinion. I'd generally recommend not using it without care. I've left it in the code because it has interesting properties in extrapolation.)The result of any of these regularizers is a system of the form B*x=0. Coupled with the interpolation system, A*x=y, we solve them in combination using a simple trick. First, I scale A and B so that each matrix has a unit 1-norm. This helps to eliminate scaling problems with the derivatives. Then I solve for the vector x such that	(norm(A*x-y))^2 + (lambda*norm(B*x))^2is minimized. Note that the parameter lambda, nominally 1 as a default in gridfit, allows the user to control the relative plate "stiffness". As lambda approaches zero, the plate becomes as flexible as possible. Stiffer plates are modeled by larger values of lambda than 1.Gridfit specifics, but in one dimensionThe above discussion was at a high level. Perhaps a specific example, solved in one dimension might help to visualize how gridfit works. I've reduced the problem to 1-d because the 2-d stuff may be more complex than we need to understand the basic ideas. In essence, I'll write a very simple piecewise linear least squares spline code. Note that I'll actually have more degrees of freedom than I have real data points in this example. What, me worry?% First, generate some random data.n = 50;x = sort(rand(n,1));% A simple function, plus some random noise.y = exp(x) + randn(n,1)/10;% Choose a set of knots/nodes for the "spline"xknots = (0:0.01:1)';% Each data points lies in some node interval, between some pair of knots.% Use histc to find out where each point lies. We will find the vector bin such that%        xknots(bin) <= x < xknots(bin+1)[junk,bin] = histc(x,xknots);% IFF we knew the values of our unknown function at each knot/node point in x, then we could% interpolate using simple linear interpolation. The problem is that we don't know those nodal% function values. We only have the data. So build a least squares problem to estimate those% unknown function values. The unknowns appear linearly, so this will be a linear least squares% problem. For example, if a data point falls exactly midway between the first two nodes in our% list of nodes, then the corresponding row for the least squares problem will look like:%        [0.5 0.5 0 0 0 0 0 0 0 0 0 0 0 0 ... ] % When you multiply this vector by our list of unknown function values at the nodes, it reduces% to 0.5*(f(1) + f(2)), which is just the linear interpolant at that point.%% So now, build the least squares problem.t = (x - xknots(bin))./(xknots(bin+1) - xknots(bin));% This does it in one line.nk = length(xknots);A = sparse(repmat((1:n)',1,2),[bin,bin+1],[1-t,t],n,nk);rhs = y;% We have now generated a sparse least squares spline problem,%%   A*f = rhs%% with 101 unknowns and 50 data points. The non-zero elements in A are seen as:spy(A)% Note that there are TWO non-zero elements in each row of A, 50 rows that correspond to 50% data points, and 101 columns, corresponding to 101 unknown function values in our grid of% nodes.% We can't solve this problem smoothly using linear algebra in Matlab, at least not yet. Try it% if you like. Here is what I get:f = A\yWarning: Rank deficient, rank = 48.f =            0            0       7.2727            0            0      0.64977       1.1866      -1.5238       10.843            0       5.0996       1.6849            0            0        3.717            0       1.0612       1.1947            0            0       1.2099            0            0            0            0            0            0       1.0186       2.4521       1.6774            0            0       12.135            0       2.4237        3.934            0       2.9862            0            0       15.946            0        3.179            0       3.5062       1.9515            0       1.4144       1.6008       9.5534            0            0            0       5.0481            0       2.4381            0       7.5175            0       1.4645       2.0286       1.8657       1.6965       2.1905            0            0            0        1.984            0        104.1       2.5044            0            0            0            0       3.7775            0            0            0       2.3394            0       2.2655       2.1796       2.2311       2.5115       11.312            0            0            0        13.56            0       2.7334            0            0            0       4.5913       1.4497       9.0807            0            0            0% Yes, this was a mess. Plot it:plot(x,y,'bo',xknots,f,'r-')% So why does gridfit work? Do you recall that I talked about a regularized solution?% Suppose we decided that the resulting curve should be everywhere "smooth"? Smoothness% is related to the second derivative of our function. So build a matrix that computes the% second derivative everywhere of our unknown function. Assuming the nodes are equally% spaced, an estimate of the second derivative at the second node looks like%%  (f(1) - 2*f(2) + f(3)) / dx^2%% At the third node, its just %%  (f(2) - 2*f(3) + f(4)) / dx^2% % Etc. So build this as a (tridiagonal) matrix. Assume the nodes are uniform.dx = xknots(2) - xknots(1);ind = (2:(nk - 1))';B = sparse(repmat(ind-1,1,3), [ind-1,ind,ind+1], repmat([1 -2 1],nk-2,1), nk-2,nk);% Convince yourself that we built B properly.spy(B)% B has the property that if we multiply it times a vector of unknown function values at the% nodes in xknots, that it computes something proportional to the second derivative at% each internal node.% Now, solve the related least squares problem,lambda = 1;f = [A; lambda*B] \ [rhs;zeros(nk-2,1)]% Plot this resultplot(x,y,'bo',xknots,f,'r-')% Note that by changing the value of lambda, we can make the curve nicely smooth.lambda = 30;f = [A; lambda*B] \ [rhs;zeros(nk-2,1)]% Plot this resultplot(x,y,'bo',xknots,f,'r-')The actual code in gridfit does something very similar, but it does it in two dimensions. Tricks with tilesWhat can you do when a gridfit problem is simply too large even for the sparse matrix abilities of matlab? This is where the tiling abilities of gridfit become valuable. What do I mean by tiling?An example might be appropriate. >> nodes = linspace(0,1,100);>> tic,zg=gridfit(rand(100,1),rand(100,1),rand(100,1),nodes,nodes);tocElapsed time is 5.814102 seconds.>> nodes = linspace(0,1,200);>> tic,zg=gridfit(rand(100,1),rand(100,1),rand(100,1),nodes,nodes);tocElapsed time is 27.118945 seconds.To solve for a 100x100 grid, gridfit must solve for 10000 unknown parameters using sparse linear algebra. A 200x200 grid is nominally 4 times as large, and it took my computer a bit more than 4x as long to solve. However, a 2000x2000 grid will probably take much more than 400 times as long to solve as a 100x100 grid. It will likely take very much longer, if it runs at all without exceeding the memory limits, due to the much slower access times for virtual memory. For example, my computer took 1020 seconds to solve a 500x500 problem.>> nodes = linspace(0,1,500);>> tic,zg=gridfit(rand(100,1),rand(100,1),rand(100,1),nodes,nodes);tocElapsed time is 1020.657834 seconds.A trick that can work, IF you have enough data to adequately populate each tile, is to break down the domain of a large grid into smaller chunks, each of which can be quickly populated by gridfit. Then gridfit composes each chunk into the whole surface. To smooth out any artifacts at the edges of each tile, gridfit allows the tiles some fractional overlap, interpolating between them in the overlapped region. Thus the tiled solution below was accomplished with essentially no dips into virtual memory, so it took far less time to generate.>> nodes = linspace(0,1,500);>> tic,zg=gridfit(rand(100,1),rand(100,1),rand(100,1),nodes,nodes,'tilesize',200,'overlap',0.2);tocElapsed time is 211.946853 seconds.Note: these random surfaces were generated purely to show speed of estimation. They are of coursepurely garbage. Also, only 100 data points may well be inadequate to estimate a tiled surface. Gridfit is not strongly dependent on the number of data points for its speed, so that number did not have an impact on the speed of estimation.
