# Supplementary material for: Fast evolution of SOS-independent multi-drug resistance in bacteria
Source: eLife. 2025 Jul 9;13:RP95058. doi: 10.7554/eLife.95058 (PMC12240585; doi:10.7554/eLife.95058)
Supplement: Source code 1. [file elife-95058-code1.zip › 0-Matlab-STORM-eLife2025/nearestneighbour/demo/html/nndemo.html]

NEARESTNEIGHBOUR Demonstration


 


# NEARESTNEIGHBOUR Demonstration

This (publishable) script nndemo.m demonstrates the features of the nearestneighbour function.

Example: Open this file in an editor (in Matlab 7+) and press publish to generate the html document

## Contents

- Summary
- Example 1: Basic nearest neighbour search
- Example 2: Nearest neighbour to subset of a point set
- Example 3: Finding nearest n neighbours
- Example 4: Finding neighbours within a specified radius
- Example 5: Combining NumberOfNeighbours and Radius
- Example 6: DelaunayMode - Large Data set, few points to lookup
- Example 7: DelaunayMode - smallish data set, lots of points to lookup

## Summary

nearestneighbour computes nearest neighbours to a set of points from a set of candidate points by Euclidean distance. Points are considered
to be matrix columns. It has a number of convenient features which might make it worth using rather than simply coding a couple-of-lines
search into an m-file. These include:

- Specifying points as either a separate matrix, or as indices into the candidate matrix
- Finding multiple neighbours
- Finding neighbours within a certain radius
- Automatically using Delaunay Triangulation if it would be faster

## Example 1: Basic nearest neighbour search

```
% 2D points of interest
P = rand(2, 5);

% Candidate point set
X = rand(2, 50);

I = nearestneighbour(P, X);

disp('Candidate points:')
disp(P)
disp('Nearest neighbours')
disp(X(:, I))

% Plot the points
plot(X(1,:), X(2,:), 'b.', P(1,:), P(2,:), 'r.', 'MarkerSize', 15);
hold on
quiver(P(1, :), P(2, :), X(1,I) - P(1, :), X(2, I) - P(2, :), 0, 'k');
hold off
```

```
Candidate points:
    0.4762    0.3942    0.1911    0.7525    0.0821
    0.2284    0.7877    0.0556    0.8136    0.5283
Nearest neighbours
    0.4680    0.3623    0.2598    0.7463    0.0811
    0.1985    0.7429    0.0139    0.7950    0.5279
```

## Example 2: Nearest neighbour to subset of a point set

Sometimes the features of interest will be certain members of a set, and you want to find the closest point(s) from within
the set

```
% Candidate 4D feature set
X = rand(4, 20);

% Find nearest neighbour to the 2nd, 4th, 10th features
I = nearestneighbour([2 4 10], X)

% Find nearest neighbours to all columns of X
I = nearestneighbour(X)
```

```
I =
    17    10     7
I =
    13    17    19    10    17     4    10    15    17     7     8    16    10     7     8    12     2    16    15     2
```

## Example 3: Finding nearest n neighbours

Let's calculate the four nearest neighbours to a point

```
% three points of interest
P = rand(2, 3);

% Candidate feature set
X = rand(2, 50);

% Calculate the 4 nearest neighbours to each point: I(:, 1) will be the 4
% nearest neighbours to P(:, 1), etc.
I = nearestneighbour(P, X, 'NumberOfNeighbours', 4)


% Plot the points - show the neighbours for one of them
plot(P(1,:), P(2, :), 'r.', X(1,:), X(2,:), 'b.', 'MarkerSize', 15)
hold on
p1 = repmat(P(1,1), 1, 4); p2 = repmat(P(2,1), 1, 4);
quiver(p1, p2, X(1, I(:, 1)) - p1, X(2, I(:, 1)) - p2, 0, 'k')
hold off
```

```
I =
    48    26    15
    23    17    31
    14    47    29
    16    46     6
```

## Example 4: Finding neighbours within a specified radius

Let's calculate the nearest neighbours to a three points within a radius of 0.2

```
% three points of interest
P = rand(2, 3);

% Candidate feature set
X = rand(2, 50);

% Calculate the nearest neighbours within a radius of 0.2 to each point:
% I(:, 1) will be the neighbours to P(:, 1), etc. Note that if different
% numbers of neighbours are found, the columns of I are padded with zeros
% at the end to make I rectangular
I = nearestneighbour(P, X, 'Radius', 0.2)

% Plot the points - show the neighbours for one of them
idx = I(I(:, 1) ~= 0, 1);
plot(P(1,:), P(2, :), 'r.', X(1,:), X(2,:), 'b.', 'MarkerSize', 15)
hold on
p1 = repmat(P(1,1), 1, length(idx)); p2 = repmat(P(2,1), 1, length(idx));
quiver(p1, p2, X(1, idx) - p1, X(2, idx) - p2, 0, 'k')
hold off
```

```
I =
    42    18    26
    15    46    17
     0    38    24
     0    31    10
     0    40     0
     0    23     0
```

## Example 5: Combining NumberOfNeighbours and Radius

Let's find at most 5 neighbours to a point within a radius of 0.2

```
% Point of interest
P = rand(2, 1);

% Candidate feature set
X = rand(2, 30);

% Calculate the 5 nearest neighbours to P, specifying that they must be
% at most 0.2 away by Euclidean distance
I = nearestneighbour(P, X, 'n', 5, 'r', 0.2);
n = length(I);

% Plot the points - show the neighbours for one of them
plot(P(1,:), P(2, :), 'r.', X(1,:), X(2,:), 'b.', 'MarkerSize', 15)
hold on

quiver(repmat(P(1), 1, n), repmat(P(2), 1, n), X(1, I) - P(1), X(2, I) - P(2), 0, 'k')
hold off
```

## Example 6: DelaunayMode - Large Data set, few points to lookup

We'll run a couple of examples and show how nearestneighbour automatically works out when to use the Delaunay triangulation. You may need to tweak the numbers of zeros to get meaningful
results on your computer. First we'll try a large data set with few points to lookup - it shouldn't use Delaunay Triangulation
as the overhead required to compute this should definitely offset any gains. We'll run nearestneighbour with DelaunayMode 'on', 'off', and 'auto'. Note that the numbers are fairly conservative to avoid crashing slow computers
with this demo!

```
% Large Data set, few points to look up
P = rand(3, 10);
X = rand(3, 5000);

fprintf('Delaunay mode test: Large data set, few candidate points:\n')

% Run with DelaunayMode off
fprintf('Elapsed Time (DelaunayMode off) : %.2g seconds\n', ...
  timingtest(P, X, 'off'))

% Run with DelaunayMode on
fprintf('Elapsed Time (DelaunayMode on)  : %.2g seconds\n', ...
  timingtest(P, X, 'on'))

% Run with DelaunayMode automatic - note 'auto' is the default
fprintf('Elapsed Time (DelaunayMode auto): %.2g seconds\n\n', ...
  timingtest(P, X, 'auto'))
```

```
Delaunay mode test: Large data set, few candidate points:
Elapsed Time (DelaunayMode off) : 0.017 seconds
Elapsed Time (DelaunayMode on)  : 0.91 seconds
Elapsed Time (DelaunayMode auto): 0.027 seconds
```

## Example 7: DelaunayMode - smallish data set, lots of points to lookup

If there are lots of points to lookup, however, using the Delaunay Triangulation should be an advantage

```
% Small Data set, lots of candidate points
P = rand(3, 10000);
X = rand(3, 500);

fprintf('Delaunay mode test: Small data set, many candidate points:\n')

% Run with DelaunayMode off
fprintf('Elapsed Time (DelaunayMode off) : %.2g seconds\n', ...
  timingtest(P, X, 'off'))

% Run with DelaunayMode on
fprintf('Elapsed Time (DelaunayMode on)  : %.2g seconds\n', ...
  timingtest(P, X, 'on'))

% Run with DelaunayMode automatic
fprintf('Elapsed Time (DelaunayMode auto): %.2g seconds\n', ...
  timingtest(P, X, 'auto'))
```

```
Delaunay mode test: Small data set, many candidate points:
Elapsed Time (DelaunayMode off) : 2.9 seconds
Elapsed Time (DelaunayMode on)  : 0.19 seconds
Elapsed Time (DelaunayMode auto): 0.19 seconds
```

Published with MATLAB® 7.3
