# Supplementary figures and images for: Fast evolution of SOS-independent multi-drug resistance in bacteria
Source: eLife. 2025 Jul 9;13:RP95058. doi: 10.7554/eLife.95058 (PMC12240585; doi:10.7554/eLife.95058)

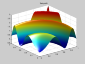

Supplement: Source code 1. [file elife-95058-code1.zip › 0-Matlab-STORM-eLife2025/gridfitdir/demo/html/gridfit_demo.png]

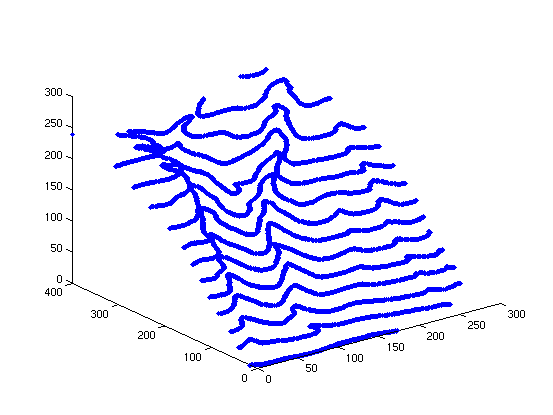

Supplement: Source code 1. [file elife-95058-code1.zip › 0-Matlab-STORM-eLife2025/gridfitdir/demo/html/gridfit_demo_01.png]

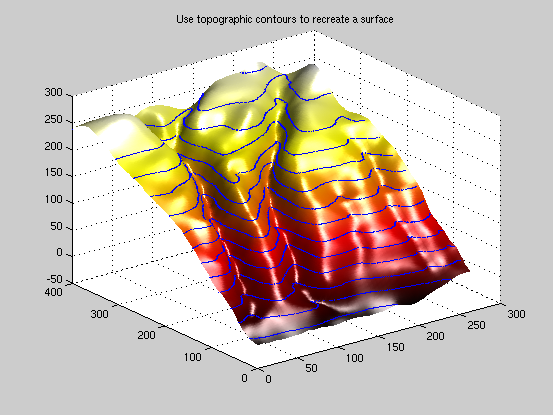

Supplement: Source code 1. [file elife-95058-code1.zip › 0-Matlab-STORM-eLife2025/gridfitdir/demo/html/gridfit_demo_02.png]

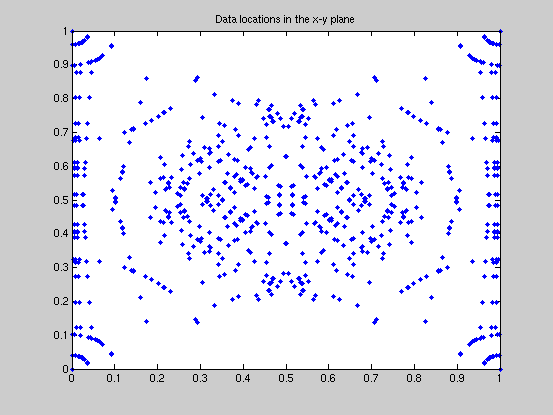

Supplement: Source code 1. [file elife-95058-code1.zip › 0-Matlab-STORM-eLife2025/gridfitdir/demo/html/gridfit_demo_03.png]

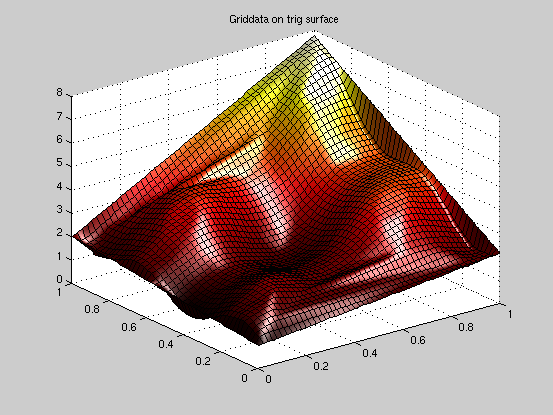

Supplement: Source code 1. [file elife-95058-code1.zip › 0-Matlab-STORM-eLife2025/gridfitdir/demo/html/gridfit_demo_04.png]

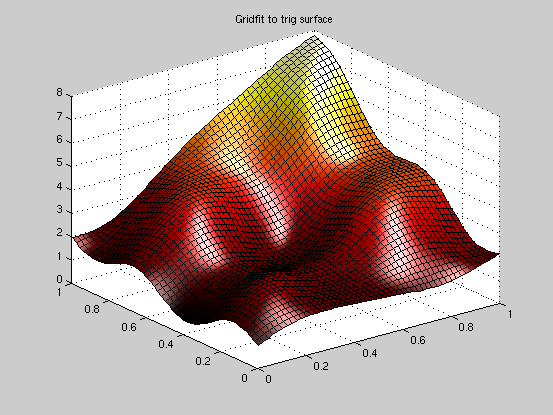

Supplement: Source code 1. [file elife-95058-code1.zip › 0-Matlab-STORM-eLife2025/gridfitdir/demo/html/gridfit_demo_05.png]

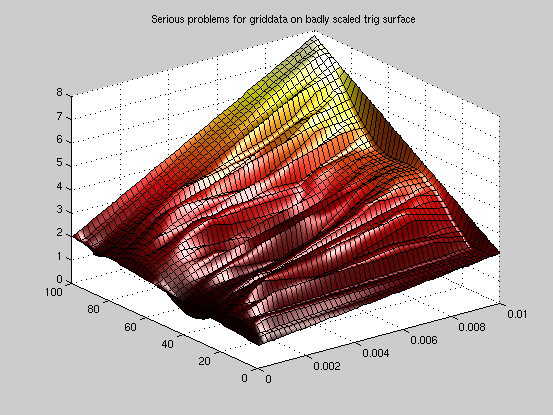

Supplement: Source code 1. [file elife-95058-code1.zip › 0-Matlab-STORM-eLife2025/gridfitdir/demo/html/gridfit_demo_06.png]

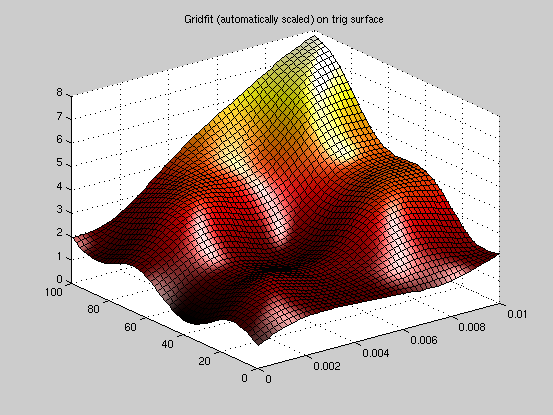

Supplement: Source code 1. [file elife-95058-code1.zip › 0-Matlab-STORM-eLife2025/gridfitdir/demo/html/gridfit_demo_07.png]

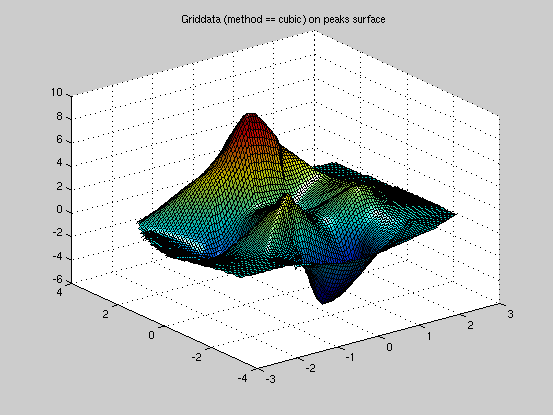

Supplement: Source code 1. [file elife-95058-code1.zip › 0-Matlab-STORM-eLife2025/gridfitdir/demo/html/gridfit_demo_08.png]

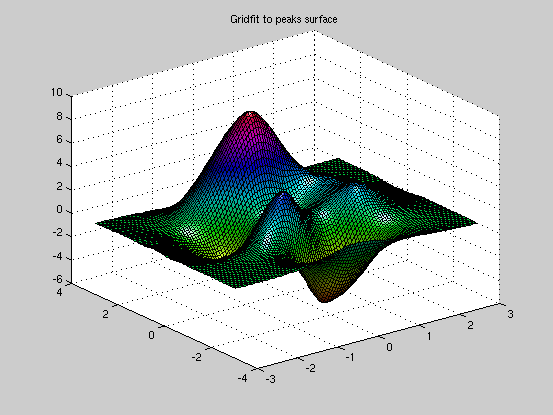

Supplement: Source code 1. [file elife-95058-code1.zip › 0-Matlab-STORM-eLife2025/gridfitdir/demo/html/gridfit_demo_09.png]

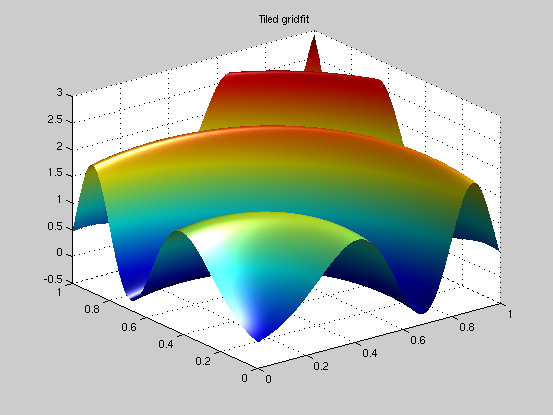

Supplement: Source code 1. [file elife-95058-code1.zip › 0-Matlab-STORM-eLife2025/gridfitdir/demo/html/gridfit_demo_10.png]

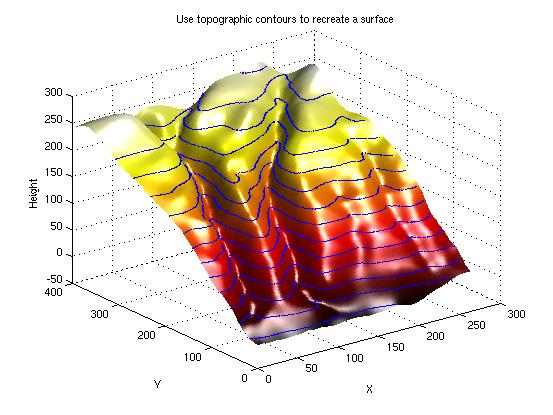

Supplement: Source code 1. [file elife-95058-code1.zip › 0-Matlab-STORM-eLife2025/gridfitdir/ravine.jpg]

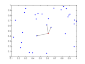

Supplement: Source code 1. [file elife-95058-code1.zip › 0-Matlab-STORM-eLife2025/nearestneighbour/demo/html/nndemo.png]

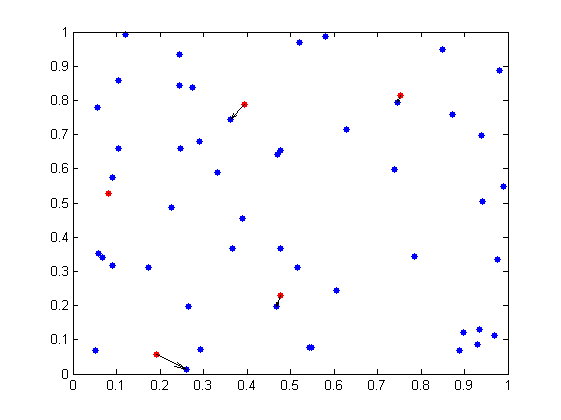

Supplement: Source code 1. [file elife-95058-code1.zip › 0-Matlab-STORM-eLife2025/nearestneighbour/demo/html/nndemo_01.png]

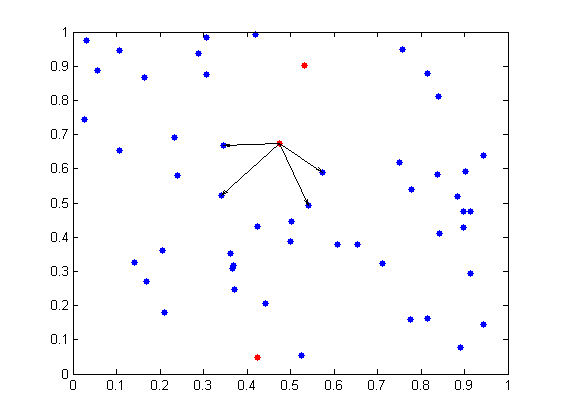

Supplement: Source code 1. [file elife-95058-code1.zip › 0-Matlab-STORM-eLife2025/nearestneighbour/demo/html/nndemo_02.png]

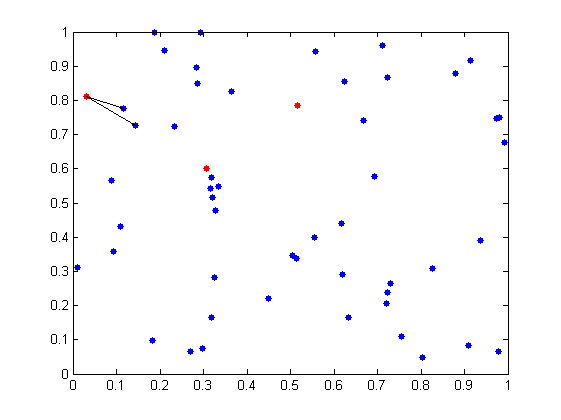

Supplement: Source code 1. [file elife-95058-code1.zip › 0-Matlab-STORM-eLife2025/nearestneighbour/demo/html/nndemo_03.png]

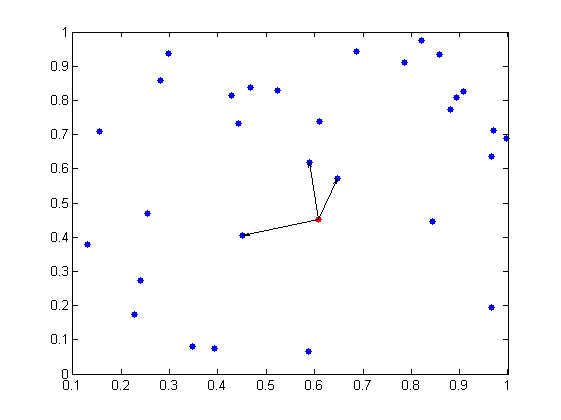

Supplement: Source code 1. [file elife-95058-code1.zip › 0-Matlab-STORM-eLife2025/nearestneighbour/demo/html/nndemo_04.png]

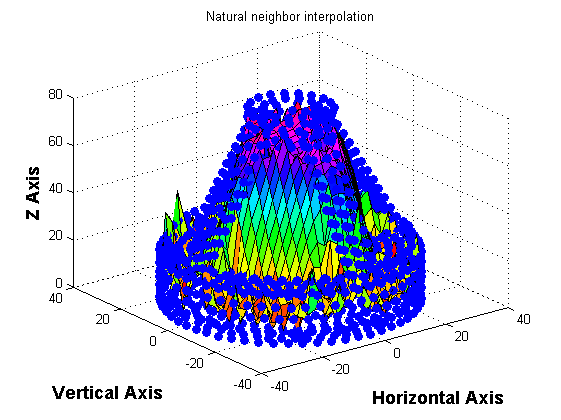

Supplement: Source code 1. [file elife-95058-code1.zip › 0-Matlab-STORM-eLife2025/plotunevenData/trimesh3ddots.png]

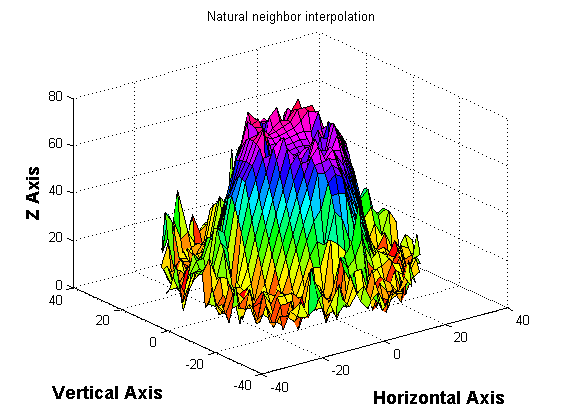

Supplement: Source code 1. [file elife-95058-code1.zip › 0-Matlab-STORM-eLife2025/plotunevenData/trimesh3dnodots.png]

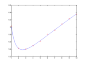

Supplement: Source code 1. [file elife-95058-code1.zip › 0-Matlab-STORM-eLife2025/PolyfitnTools/demo/html/polyfitn_demo.png]

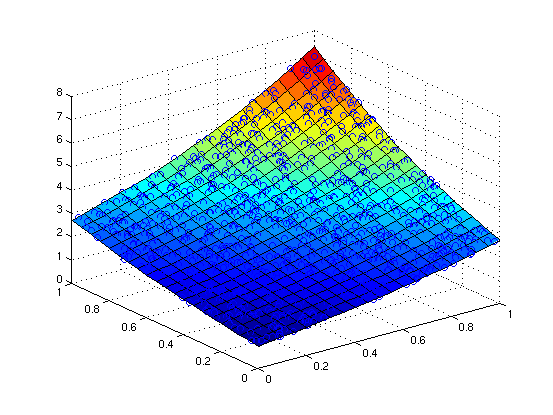

Supplement: Source code 1. [file elife-95058-code1.zip › 0-Matlab-STORM-eLife2025/PolyfitnTools/demo/html/polyfitn_demo_01.png]

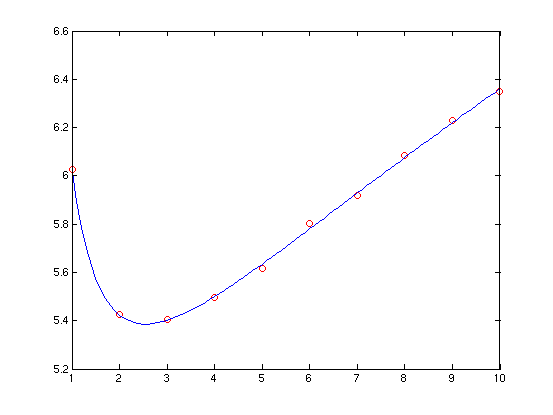

Supplement: Source code 1. [file elife-95058-code1.zip › 0-Matlab-STORM-eLife2025/PolyfitnTools/demo/html/polyfitn_demo_02.png]
